# Gut Microbiome Alterations Affect Glioma Development and Foxp3 Expression in Tumor Microenvironment in Mice

**DOI:** 10.3389/fonc.2022.836953

**Published:** 2022-03-08

**Authors:** Yiqi Fan, Qing Su, Junxiao Chen, Yong Wang, Shuai He

**Affiliations:** Department of Pharmacy, Zhujiang Hospital, Southern Medical University, Guangzhou, China

**Keywords:** glioma, gut microbiome, tumor microenvironment, antibiotics, fecal microbiota transplantation

## Abstract

Glioma is the most common malignant tumor of the central nervous system (CNS), with high degree of malignancy and poor prognosis. The gut microbiome (GM) is composed of microorganisms with different properties and functions, which play an important role in human physiology and biological activities. It has been proved that GM can affect the development of glioma through natural immunity, but whether GM can affect glioma through adaptive immunity and whether there are some microorganisms in the GM that may affect glioma growth still remain unclear. In our study, we evaluated the relationship between GM and glioma. We proved that (I) glioma growth can induce structural changes of mouse GM, including the decreased abundance of *Bacteroidia* and increased abundance of *Firmicutes*. (II) GM dysbiosis can downregulate Foxp3 expression in the brain and promote glioma growth. A balanced environment of GM can upregulate the expression of Foxp3 in the brain and delay the development of glioma. (III) The increased abundance of *Bacteroidia* is associated with accelerated glioma progression, while its decreased abundance is associated with delayed glioma progression, which may be one of the key microorganisms affecting glioma growth. This study is helpful to reveal the relationship between GM and glioma development and provide new ideas for adjuvant therapy of glioma.

## Introduction

Glioma is the most common malignant tumor of the central nervous system (CNS), with a high recurrence rate and poor prognosis due to its invasiveness and immune escape (1, 2). At present, the main treatment for glioma is surgery combined with high-precision chemoradiotherapy and tumor treatment field (TTF) (3). Glioma has made advances in chemotherapy and surgery, but overall survival (OS) has not increased significantly, and the challenges of rapid spread, molecular heterogeneity, and drug delivery across the blood–brain barrier limit the therapy effectiveness (4, 5). As immunotherapy is gradually applied to malignant tumors, the study of the tumor microenvironment and tumor–host interaction is helpful to develop new therapeutic strategies, which is an important direction to explore potential therapies for glioma (6).

The human gut microbiome (GM) consists of microorganisms with different properties and functions. In recent years, more and more evidence supports the role of the gut microbiome in human physiology and biological activities. Gut microbiome dysbiosis is an unbalanced bacterial environment, which can induce an inflammatory environment and immune suppression and affect central nervous system diseases such as stroke, autoimmune encephalomyelitis, and multiple sclerosis (MS) (7–11). Studies have shown that GM is sensitive to cancer, which plays an important role in the development of melanoma, breast cancer, colorectal cancer, lung cancer, and other malignant tumors and affects tumor immunotherapy (12–17).

Based on available information, we speculated that there was also a certain connection between GM and glioma, and GM could affect the growth of glioma in some way. Giuseppina Alessandro et al. found the relationship between natural immunity and glioma that the change of GM can affect NK cell subsets and effector functions in the brain, bone marrow, and spleen to affect glioma development (18). However, it is not clear whether GM can regulate glioma development by influencing adaptive immunity. At present, the targets of glioma immunotherapy mostly focus on tumor microenvironment components. Whether GM has a regulatory effect on some immune components in the glioma microenvironment to affect the development of glioma is an important target of our research, and the results will be significant to the adjuvant therapy of glioma.

In this study, we (I) investigated the effects of glioma growth on gut microbiome in mice, (II) studied the effects of gut microbiome dysbiosis on glioma development and the expression of CD8 and Foxp3 in tumor microenvironment, and (III) evaluated the role of a balanced gut microbiome microenvironment in glioma development.

## Materials and Methods

### Cell Culture

The mouse glioblastoma cell line GL261-Luc was obtained from our laboratory and cultured in DMEM supplemented with 10% FBS in a humidified 5% CO_2_ cell incubator at 37°C.

### Animals

We used three batches of specific-pathogen-free (SPF) male and female C57BL/6 mice. SPF male and female C57BL/6 mice (6–8 weeks) were purchased from Zhuhai BesTest Bio-Tech Co., Ltd. (Zhuhai, China). All mice were housed in an SPF environment in the Animal Experimental Center of Zhujiang Hospital at Southern Medical University (Guangzhou, China) where the temperature was maintained at 25°C with a 12-h light/dark cycle and free access to food and water. After adaptive feeding for 7 days, mice were randomly allocated to the following groups. All experiments were approved by the Animal Ethics Committee of Zhujiang Hospital of Southern Medical University (No. LAEC-2021-008). All animal experiments are conducted in accordance with animal ethics.

The first batch of mice (n = 5): GL261-Luc cells were implanted into mice’s brain on day 1. Tumor development was monitored by *in vivo* optical imaging technology. Fecal samples were collected with sterile tubes from mice 1 day before tumor implantation (1st sample), at 14 days of tumor growth (2nd sample), and at 21 days of tumor growth (3rd sample) and stored at -80°C.

The second batch of mice (n = 10): mice were randomly allocated to two groups (ABT (n = 5): the group with antibiotic treatment. Non-ABT (n = 5): the group without antibiotic treatment). ABT (antibiotic-treated): ampicillin 0.5 mg/ml, vancomycin 0.25 mg/ml, neomycin 0.5 mg/ml, and metronidazole 0.5 mg/ml were added into mice’s drinking water. The drug administration continued in the period of the experiment. ABT and Non-ABT solutions were changed once a week. GL261-Luc cells were implanted into mice’s brain on day 7. Tumor development was monitored by *in vivo* optical imaging technology. Fecal samples were collected with sterile tubes from mice 1 day before tumor implantation (1st sample) and at 21 days of tumor growth (2nd sample) and stored at -80°C.

The third batch of mice (n = 10): mice were randomly allocated to two groups (ABT+FMT (n = 5): fecal microbiota transplantation after antibiotic treatment. ABT+NaCl (n = 5): intragastric administration with physiological saline after antibiotic treatment). ABT (antibiotic-treated): ampicillin 0.5 mg/ml, vancomycin 0.25 mg/ml, neomycin 0.5 mg/ml, and metronidazole 0.5 mg/ml were added into mice’s drinking water. The drug administration lasted 7 days. Fresh fecal samples of healthy C57BL/6 mice were collected with sterile tubes, and glycerin was added before freezing. The final concentration was kept at 10%, and then the healthy mouse fecal samples were stored at -80°C. On the day of use, the fecal samples were thawed in a 37°C water bath, dissolved in a certain volume of normal saline, made into 400 mg/ml fecal microbiota transplantation (FMT) suspension, and transplanted into ABT+FMT mice within 6 h. FMT: after ABT, the gut microbiome was balanced for 24 h and FMT was performed. Before FMT, mice were fasted for 30 min, administered gavage of 100 μl 1 M NaHCO_3_ to buffer stomach pH, and then administered gavage of 150 μl to balance the gut microbiome. 24 h later, mice were given FMT, 80–120 μl/mouse, once a day, 7 days in total. After FMT, fecal samples of mice were collected by sterile tubes and stored at -80°C, and 16S rDNA was used to detect the gut microbiome. 24 hours after FMT, GL261-Luc cells were implanted into mice’s brain. Tumor development was monitored by *in vivo* optical imaging technology.

### 16s rDNA Gene Sequencing

Stool samples were extracted with the QIAamp Fast DNA Stool Mini Kit. 16S rDNA gene sequencing was carried out in the Division of Laboratory Medicine of Zhujiang Hospital of Southern Medical University.

### *In Vivo* Optical Imaging Technology

The tumor progression was monitored by *in vivo* optical imaging technology at 3, 9, 15, and 21 days of tumor growth. The head hair of mice was removed 1 day before imaging. D-Luciferin, potassium salt working solution (AAT Bioquest^®^, Inc., 12507 D-Luciferin, potassium salt *UltraPure Grade*) was intraperitoneally injected into mice at 15 min before living imaging. Animals were anesthetized by the XGI-8 Gas Anesthesia System. Then, mice were imaged in the IVIS^®^ Spectrum Imaging System and analyzed by Living Image^®^ Software 4.4 for IVIS^®^ Spectrum.

### Hematoxylin–Eosin Staining and Immunohistochemistry

Hematoxylin–eosin staining (HE) staining was used to assess tumor cells. In brief, the brain sections were prepared, the cell nuclei were stained with hematoxylin dye, and the cytoplasm was sealed with eosin dye. Section dehydration, slice sealing, microscopic examination, image collection, and analysis were performed.

Immunohistochemical staining was used to evaluate the expression of CD8 and Foxp3 in mouse brain tissues. In brief, brain sections were prepared, 3% hydrogen peroxide (Boster Biological Technology Co., Ltd., Pleasanton, CA, USA, SA1022) was used for the removal of endogenous peroxidase, and 5% BSA (Boster Biological Technology Co., Ltd., SA1022) was used for the antigen closing of tissue sections and incubation at 4°C overnight with anti-CD8 (Cohesion Biosciences, Gandhinagar, India, CQA6570) and anti-FOXP3 (Cohesion Biosciences CQA3716). Then, biotin-labeled goat anti-rabbit IgG secondary antibody (Boster Biological Technology Co., Ltd., SA1022) was cultured at room temperature for 1 h, treated with SABC (Boster Biological Technology Co., Ltd., SA1022), and incubated at room temperature for 1 h. The DBA Chromogenic Kit was used for staining (Boster Biological Technology Co., Ltd., AR1027) and dying again with hematoxylin. 75%-100% ethanol was used for dehydration, xylene was used for transparency, and neutral gum was used for sealing the slices. Finally, images were taken with the microscope.

### Statistical Analysis

All results are expressed as means ± SE of at least three independent experiments unless stated otherwise. Every sample contributed to analysis. IBM SPSS Statistics 20 was used for statistical analysis. The independent-sample T-test was used for statistical analysis, and p < 0.05 means the difference is statistically significant.

## Results

### Development of Glioma Induces Changes in Mouse Gut Microbiome

**Figure 1:**
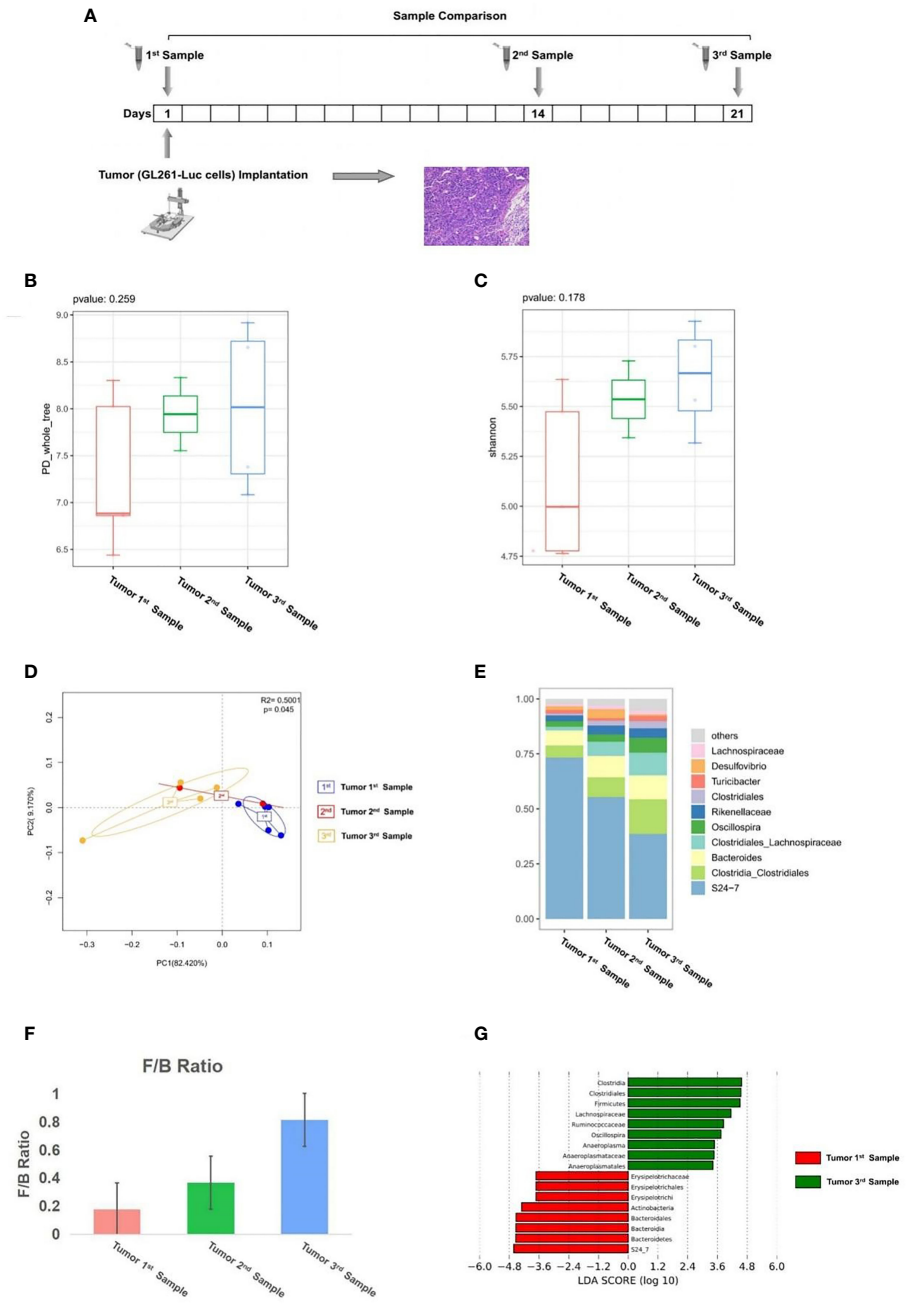
**(A)** Experimental design and stool sample collection (first part). **(B–G)** Glioma growth induced structural changes of gut microbiome in mice. Comparison of tumor 1st to 3rd samples (n = 5). **(B)** The PD_whole_tree alpha diversity index. **(C)** The Shannon alpha diversity index. **(D)** PCoA plot of β-diversity. **(E)** Taxa analysis. **(F)** *Firmicutes* to *Bacteroides* ratio. **(G)** Bar plot of LDA score from LEfSe analysis.

First, to evaluate whether glioma growth can change the GM structure, we implanted GL261-Luc cells into healthy C57BL/6 mice to establish glioma models, and then we performed a comparison of samples before tumor implantation (1st sample), samples in 14 days of tumor growth (2nd sample), and samples in 21 days of tumor growth (3rd sample) (**Figure 1A**). From this comparison, the PD_whole_tree (p = 0.259) and Shannon index (p = 0.178) showed similar bacterial community richness and diversity (**Figures 1B, C**), while the principal coordinate analysis (PCoA) of weighted pairwise Bray–Curtis distances demonstrated differential clustering patterns (p = 0.045) (**Figure 1D**). Taxa analysis showed that before tumor cell implantation, the GM of mice was mainly composed of *Bacteroidetes*, *Firmicutes*, and *Actinobacteria* (**Figure 1E**). During the growth of glioma, the abundance of *Bacteroidia* and *Actinobacteria* decreased, while the abundance of *Firmicutes* increased (**Figure 1E**), which was indicated by the F/B ratio (**Figure 1F**). LEfSe analysis showed that the abundance of *Bacteroidetes*, which dominated by *S24-7*, decreased in the development of tumor, and the abundance of *Firmicutes* such as *Clostridia_Clostridiales*, *Clostridiales_Lachnospiraceae*, and *Oscillospira* increased during tumor growth (**Figure 1G**).

Our results suggest that the abundance of *Bacteroidia* and *Actinobacteria* in mice GM decreased with the growth of glioma, while the abundance of *Firmicutes* increased with the development of glioma. The results showed that glioma development induced structural changes of gut microbiome in mice.

### Gut Microbiome Dysbiosis Downregulates Foxp3 Expression in Brain and Promotes Glioma Growth

**Figure 2:**
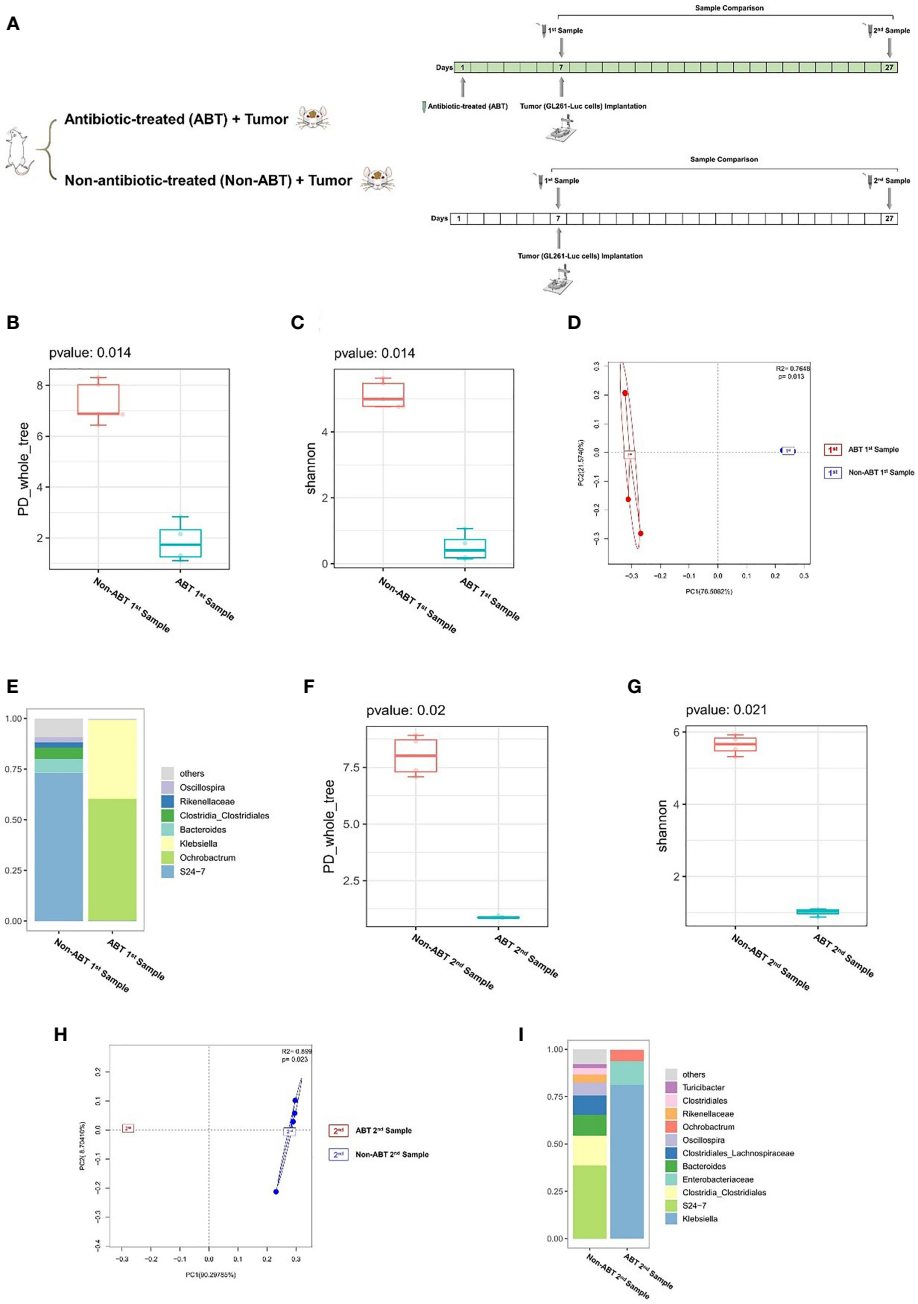
**(A)** Experimental design and stool sample collection (second part). **(B–I)** Combined antibiotic treatment resulted in dysbiosis of the gut microbiome in mice. **(B–E)** Comparison between antibiotic-treated (ABT) 1st sample and non-antibiotic-treated (Non-ABT) 1st samples (n = 5). **(F–I)** Comparison between antibiotic-treated (ABT) 2nd sample and non-antibiotic-treated (Non-ABT) 2nd samples (n = 5). **(B)** The PD_whole_tree alpha diversity index of the first sample. **(C)** The Shannon alpha diversity index of the first sample. **(D)** PCoA plot of β-diversity of the first sample. **(E)** Taxa analysis of the first sample. **(F)** The PD_whole_tree alpha diversity index of the second sample. **(G)** The Shannon alpha diversity index of the second sample. **(H)** PCoA plot of β-diversity of second sample. **(I)** Taxa analysis of the second sample.

Previous experiments proved that glioma growth induced structural changes of GM in mice, but the role of GM in the development of glioma remains unknown. In order to investigate the effect of GM on glioma development, we added compound antibiotics containing ampicillin, vancomycin, neomycin, and metronidazole into drinking water of some mice and continued the drug administration in the experiment. After 7 days of the combined antibiotic administration, we performed a comparison of antibiotic-treated (ABT) mouse and non-antibiotic-treated (Non-ABT) mouse fecal samples (1st sample) to evaluate the effect of antibiotics on the gut microbiome (**Figure 2A**). From this comparison, the PD_whole_tree and Shannon index (p = 0.014) showed significantly different bacterial community richness and diversity (**Figures 2B, C**). Moreover, the principal coordinate analysis (PCoA) of weighted pairwise Bray–Curtis distances demonstrated differential clustering patterns (p = 0.013) (**Figure 2D**). Taxa analysis showed that after 7 days of antibiotic treatment, the abundance of *Bacteroides* and *Firmicutes* in the GM of ABT mice was significantly decreased, and the GM was dominated by *Proteobacteria* such as *Ochrobactrum* and *Klebsiella* (**Figure 2E**). The GM of Non-ABT mice was mainly composed of *Bacteroidetes*, *Firmicutes*, and *Actinobacteria*, and the abundance of *S24-7* was the highest, followed by *Bacteroides* and *Clostridia_Clostridiales* (**Figure 2E**). These results suggest that ABT mice have established a gut microbiome dysbiosis environment.

**Figure 3:**
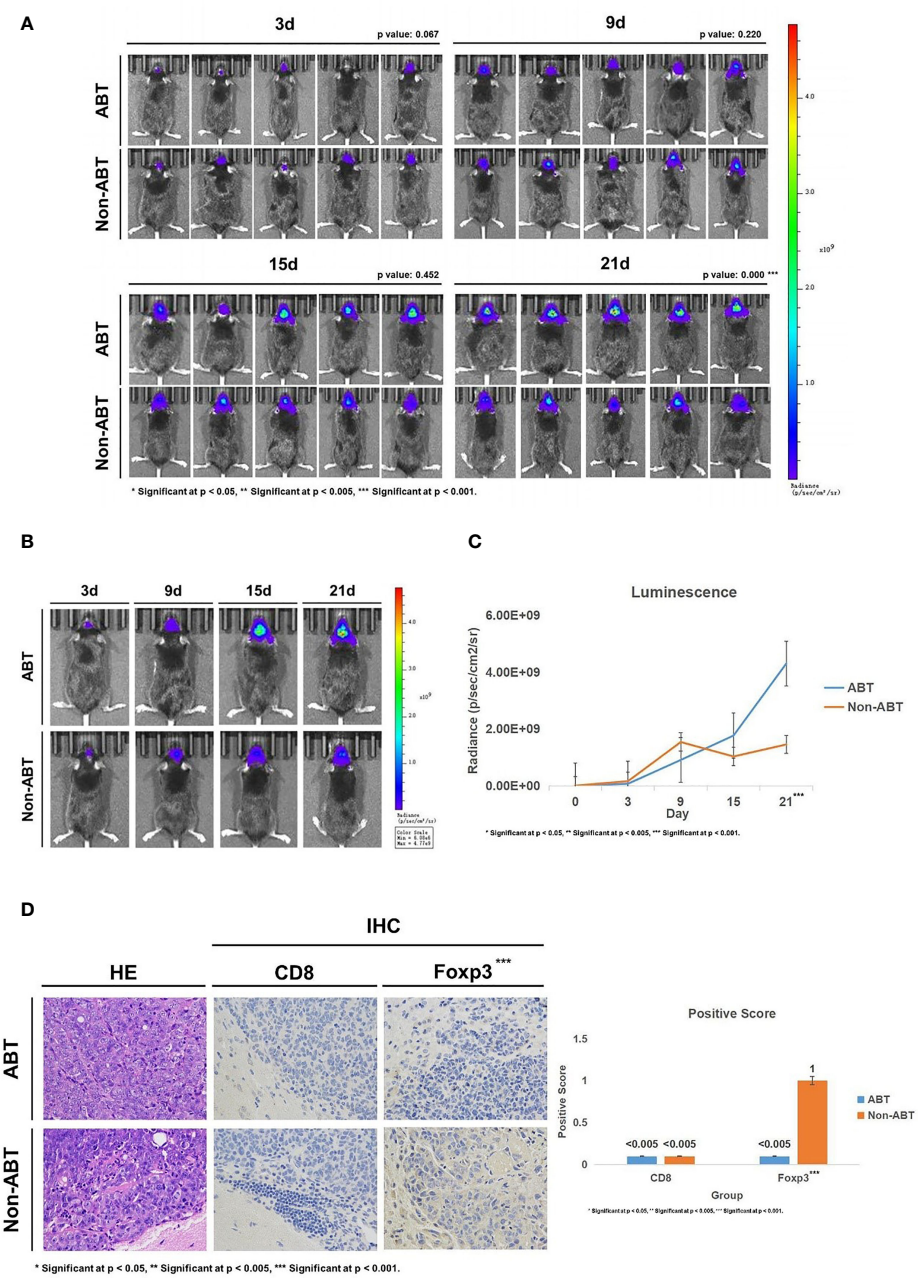
**(A)** Living image results of glioma tumor growth in mice (n = 5). The living image was performed on mice at 3, 9, 15, and 21 days of glioma growth. ABT: the group with antibiotic treatment. Non-ABT: the group without antibiotic treatment. **(B)** Living image results of glioma tumor growth in mice (n = 1). Living image was performed on mice at 3, 9, 15, and 21 days of glioma growth. ABT: the group with antibiotic treatment. Non-ABT: the group without antibiotic treatment. **(C)** Luminescence curve of glioma in living image (n = 5). ABT: the group with antibiotic treatment. Non-ABT: the group without antibiotic treatment. **(D)** The expressions of CD8 and Foxp3 in the ABT group and non-ABT group were detected by immunohistochemistry (×400 magnification). ABT: the group with antibiotic treatment. Non-ABT: the group without antibiotic treatment. ***Significant at p < 0.001

Subsequently, we implanted GL261-Luc cells into ABT and Non-ABT C57BL/6 mice to establish glioma models (**Figure 2A**). The tumor progression was monitored by living image at 3, 9, 15, and 21 days of tumor growth to evaluate the impact of gut microbiome dysbiosis on glioma development (**Figures 3A–C**). The results showed that compared with the Non-ABT mice, the tumor deterioration of ABT mice became worse after 15 days of tumor cell implantation (**Figures 3A–C**). After 21 days of tumor cell implantation, there was a significant difference in tumor growth between ABT mice and Non-ABT mice (p = 0.004) (**Figure 3C**). These results suggest that gut microbiome dysbiosis can worsen glioma progression.

Next, we detected the expression of CD8 and Foxp3 in mouse brain tissues to explore the effect of gut microbiome dysbiosis on the glioma microenvironment. Immunohistochemical results showed that Foxp3 expression was lower in the ABT group than in the Non-ABT group, while CD8 expression was not significantly different between the Non-ABT group and the ABT group (**Figure 3D**). These results suggest that gut microbiome dysbiosis may promote glioma growth by downregulating Foxp3 expression in the glioma microenvironment without affecting the CD8 expression level.

In addition, we performed a comparison of ABT mouse and Non-ABT mouse fecal samples at 21 days of tumor growth (2nd sample) to evaluate the sustaining effect of antibiotics on GM. From this comparison, the PD_whole_tree (p = 0.020) and Shannon index (p = 0.021) showed significantly different bacterial community richness and diversity (**Figures 2F, G**). Moreover, the principal coordinate analysis (PCoA) of weighted pairwise Bray–Curtis distances demonstrated differential clustering patterns (p = 0.023) (**Figure 2H**). Taxa results showed that after 21 days of tumor growth, the main components of the GM in ABT mice were *Proteobacteria* including *Klebsiella*, *Ochrobactrum*, and *Enterobacteriaceae* (**Figure 2I**). The abundance of *S24-7* in the GM of Non-ABT mice was decreased, but *S24-7*, *Bacteroides*, and *Clostridia_Clostridiales* were still the main components of bacterial community (**Figure 2I**). These results suggested that the GM of ABT mice maintained gut microbiome dysbiosis during tumor growth under the continuous administration.

Overall, our results suggest that gut microbiome dysbiosis may downregulate Foxp3 expression in mouse brain to promote glioma growth.

### Maintaining Gut Microbiome Environment Balance Upregulates Foxp3 Expression in Brain and Slow Down Glioma Growth

**Figure 4:**
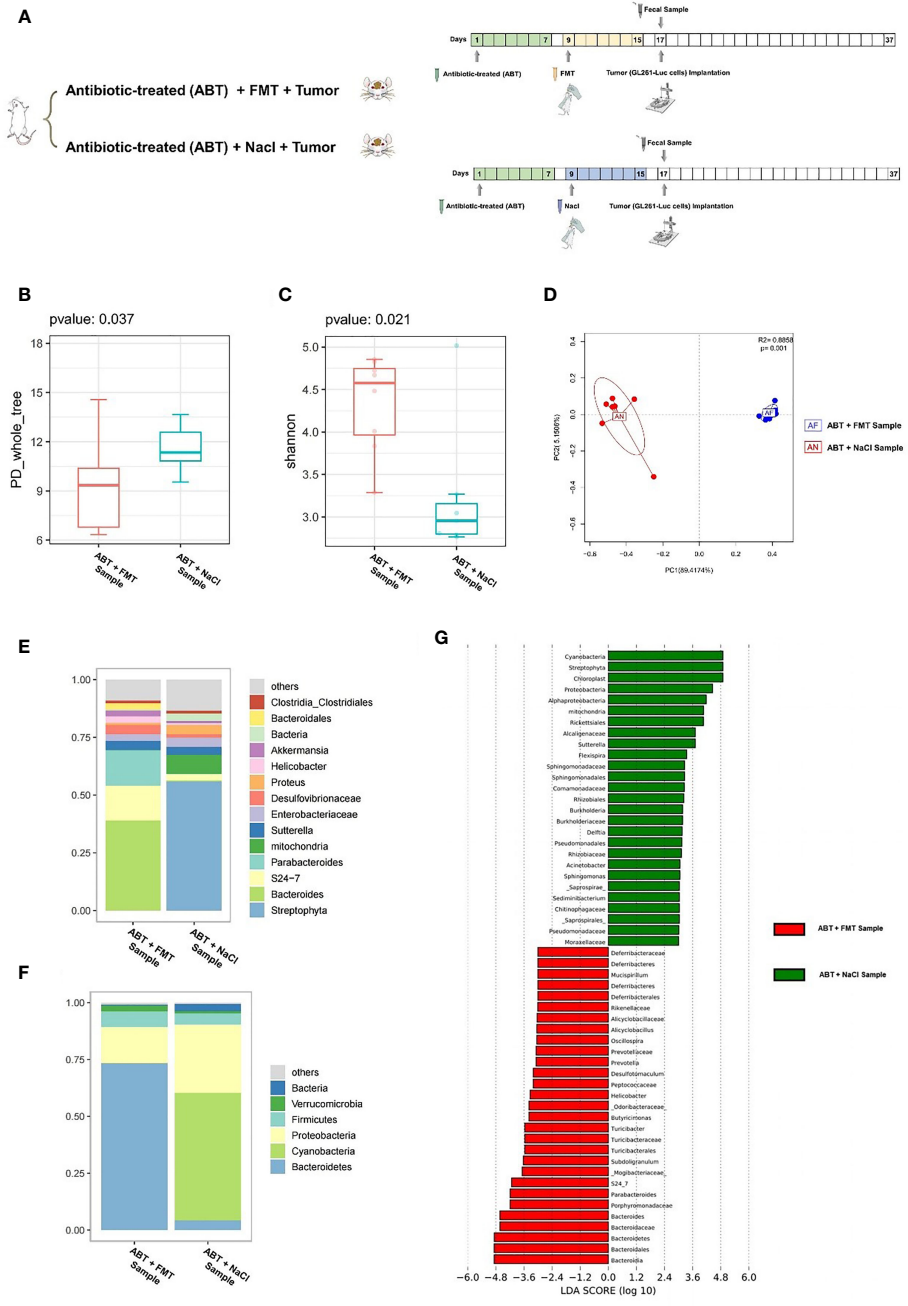
**(A)** Experimental design and stool sample collection (third part). **(B–G)** Comparison between the sample of fecal microbiota transplantation after antibiotic treatment (ABT+FMT) and the sample of intragastric administration with physiological saline after antibiotic treatment (ABT+NaCl). **(B)** The PD_whole_tree alpha diversity index. **(C)** The Shannon alpha diversity index. **(D)** PCoA plot of β-diversity. **(E)** Taxa analysis for genus. **(F)** Taxa analysis for phylum. **(G)** Bar plot of the LDA score from LEfSe analysis.

In order to exclude the effects of antibiotics on glioma growth, we gave healthy C57BL/6 mice with compound antibiotic drinking water for 7 days before tumor cell implantation and then implanted healthy mouse fecal samples into some mice which received antibiotic treatment. Next, we performed a comparison of ABT+FMT (fecal microbiota transplantation after antibiotic treatment) mice and ABT+NaCl (intragastric administration with physiological saline after antibiotic treatment) mouse fecal samples before tumor cell implantation to evaluate the gut microbiome structure (**Figure 4A**). From this comparison, the PD_whole_tree (p = 0.037) and Shannon index (p = 0.021) showed significantly different bacterial community richness and diversity (**Figures 4B, C**). Moreover, the principal coordinate analysis (PCoA) of weighted pairwise Bray–Curtis distances demonstrated differential clustering patterns (p = 0.001) (**Figure 4D**). Taxa analysis showed that *Bacteroidetes* were supplemented in the GM of ABT+FMT mice. Compared with ABT+NaCl mice, the abundance of *Bacteroidetes* in the GM of ABT+FMT mice was significantly increased (**Figure 4F**). On 7 days of fecal microbiota transplantation (FMT), the main components in GM of ABT+FMT mice were *Bacteroides*, *S24-7*, and *Parabacteroides*, while the main components in GM of ABT+NaCl mice were *Proteus*, *Enterobacteriaceae*, and *Sutterella* (**Figures 4E, F**). LEfSe analysis showed that the abundance of *Bacteroidetes* in ABT+FMT mice was the highest, while the abundance of *Proteobacteria*, *Rickettsiales*, *Alcaligenaceae*, *Sutterella*, and *Flexispira* in the ABT+NaCl group was higher (**Figure 4G**). The above results suggested that there was a significant difference in GM structure between the two groups of mice before tumor cell implantation. The abundance of *Bacteroidetes* in the GM of ABT+FMT mice was significantly increased, which indicated that the gut microbiome environment tended to balance, while the GM of ABT+NaCl mice was in a state of dysbiosis.

**Figure 5:**
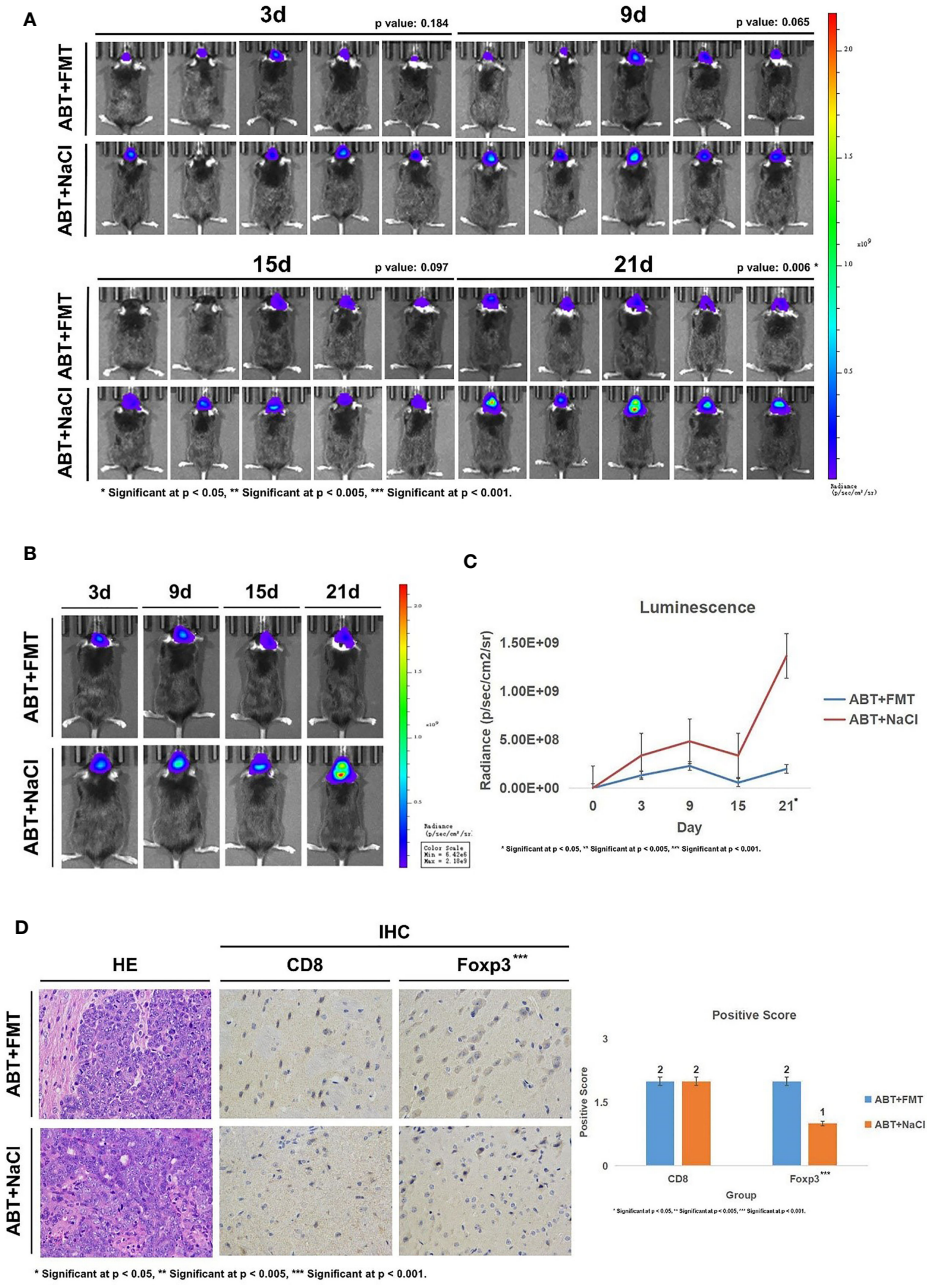
**(A)** Living image results of glioma tumor growth in mice (n = 5). Living image was performed on mice at 3, 9, 15, and 21 days of glioma growth. ABT+FMT: fecal microbiota transplantation after antibiotic treatment. ABT+NaCl: intragastric administration with physiological saline after antibiotic treatment. **(B)** Living image results of glioma tumor growth in mice (n = 1). Living image was performed on mice at 3, 9, 15, and 21 days of glioma growth. ABT+FMT: fecal microbiota transplantation after antibiotic treatment. ABT+NaCl: intragastric administration with physiological saline after antibiotic treatment. **(C)** Luminescence curve of glioma in living image (n = 5). ABT+FMT: fecal microbiota transplantation after antibiotic treatment. ABT+NaCl: intragastric administration with physiological saline after antibiotic treatment. **(D)** The expressions of CD8 and Foxp3 in the ABT+FMT group and ABT+NaCl group were detected by immunohistochemistry (×400 magnification). *Significant at p < 0.05, **Significant at p < 0.005, ***Significant at p < 0.001

Subsequently, we implanted GL261-Luc cells into ABT+FMT and ABT+NaCl C57BL/6 mice to establish glioma models (**Figure 4A**). The tumor progression was monitored by living image at 3, 9, 15, and 21 days of tumor growth to evaluate the impact of a balanced gut microbiome environment on glioma development (**Figures 5A–C**). The results showed that the tumor development rate of mice in ABT+FMT mice was significantly lower than that in ABT+NaCl mice (**Figures 5A–C**). After 21 days of tumor growth, there was a significant difference in tumor growth between ABT+FMT mice and ABT+NaCl mice (p = 0.018) (**Figure 5C**). These results suggest that maintaining a balanced gut microbiome environment can slow down the development of glioma.

Next, we detected CD8 and Foxp3 expression in brain tissues of ABT+FMT and ABT+NaCl mice. Immunohistochemical results showed that there was no significant difference in CD8 expression between ABT+FMT mice and ABT+NaCl mice (**Figure 5D**). However, the expression of Foxp3 in ABT+FMT mice was higher than that in ABT+NaCl mice (**Figure 5D**). These results suggest that maintaining the balance of the gut microbiome environment can upregulate Foxp3 expression in mouse brain.

Overall, our results suggest that maintaining a balanced and stable gut microbiome environment in mice can upregulate Foxp3 expression in the brain to slow down the development of glioma.

## Discussion

In this study, we investigated the relationship between GM and glioma. Then, we evaluated the effect of GM on the expression of CD8 and Foxp3 in the glioma microenvironment.

First, to prove that glioma growth induced changes in GM of mice, we collected fecal samples from mouse models in the period of glioma development for 16S rDNA detection. The results showed that the development of glioma in mice led to a decrease in the abundance of *Bacteroidetes*, which was mainly composed of *S24-7*, and an increase in the abundance of *Firmicutes* including *Clostridia_Clostridiales*, *Clostridiales_Lachnospiraceae*, and *Oscillospira*. Anthony Patrizz and Xiao-Chong Li et al. observed that glioma growth led to biological dysbiosis of GM in mice, with the most significant changes in *Bacteroidetes* and *Firmicutes (* 19, 20), which was consistent with our results.

We also demonstrated that antibiotic treatment led to gut microbiome dysbiosis, downregulated the Foxp3 expression in mouse brain, and promoted glioma development in mice. Maintaining the balance of GM in mice can upregulate Foxp3 expression in brain and slow down glioma growth.

Foxp3 is a member of the forkhead-type transcription factor family, which is widely regarded as a specific molecular marker for regulatory cells (Tregs). However, Foxp3 expression is not limited to Tregs but is also expressed in different types of tumor cells, including glioma cells (21). Studies have shown that Foxp3 is highly expressed in thyroid carcinoma and pancreatic cancer, while no or weak Foxp3 expression was detected in the normal cells from these tumor cells, suggesting that Foxp3 has tumor-promoting functions (22, 23). However, the malignant transformation of breast cancer is associated with the loss of Foxp3 expression, suggesting that Foxp3 may be a tumor suppressor (24). These results suggest that Foxp3 may play a dual role as an activator or suppressor in different types of tumor cells. Janka Have-Feindt et al. demonstrated that overexpression of Foxp3 in glioma cells facilitates apoptosis and increases cell sensitivity to apoptotic stimuli (25). In addition, Biao Zhang et al. found that downregulation of Foxp3 can promote the growth and inhibit the apoptosis of glioma cells, while upregulation of Foxp3 can inhibit the invasion and migration of glioma cells, suggesting that Foxp3 may inhibit the proliferation, migration, and invasion of glioma cells (21). These results suggest that Foxp3 plays an important role in the growth of glioma as a tumor suppressor. In our second experiment of mice, we found that Foxp3 was highly expressed in the brain of Non-ABT mice, but not in ABT mice, indicating that the glioma growth rate of Non-ABT mice was relatively slower than that of ABT mice, which was consistent with the results of living image. In our third experiment of mice, the gut microbiome environment of ABT+FMT mice approached balance, and the expression of Foxp3 in the brain was upregulated, suggesting that the tumor growth of ABT+FMT mice was relatively slow, which was also consistent with our living imaging results. Therefore, we believe that GM can affect glioma growth by affecting Foxp3 expression in the glioma microenvironment.

The role of the gut microbiome in the immune system has also been well studied in germ-free (GF) animals. GF animals have severe immune deficiencies, which are manifested as defects in lymphoid structure and immune function, demonstrating that the GM is essential for the activation and function of immune cells (16, 26). In addition, Giuseppina D. Alessandro et al. demonstrated in mouse models that antibiotic-induced dysbiosis can induce NK cell damage and change the phenotype of microglia to promote the growth of glioma, indicating that gut microbiome dysbiosis can affect the progression of glioma by affecting natural immunity (18). Eiji Miyauchi et al. found in the study of dysbiosis and autoimmune diseases that the number of CD4+T cells in the small intestine of antibiotic-treated mice was significantly increased, indicating the correlation between GM and adaptive immunity (11). Our results have proved that the gut microbiome dysbiosis in mice can lead to the decrease of Foxp3 expression in the glioma microenvironment and promote the development of glioma, further indicating that GM have a certain effect on adaptive immunity.

This evidence indicates the role of the gut–immune–brain axis in glioma development. However, the regulatory mechanism of Foxp3 in tumor cells remains unclear (27). Therefore, further experiments are needed to explore how GM affects glioma development by regulating Foxp3 expression.

In our study, we found that with the development of glioma, the abundance of *Bacteroidia* decreased significantly. Under the condition of gut microbiome dysbiosis, the abundance of *Bacteroidia* was close to zero, and the deterioration of glioma was aggravated. After fecal microbiota transplantation, the abundance of *Bacteroidia* in the GM of ABT+FMT mice was supplemented, and the growth rate of glioma was slowed down. We speculated that the decreased abundance of *Bacteroidia* might be one of the reasons for the deterioration of glioma.

*Bacteroides* is a kind of Gram-negative bacteria, which usually exists in the human intestine, mouth, and other parts (28). Many studies have linked *Bacteroides* with the development of inflammatory bowel disease (IBD). Round J. L. et al. found that *Bacteroides* can express polysaccharide A and affect the secretion of protective cytokines to resist colitis (29, 30). Edda Russo et al. found that for inflammatory bowel disease patients receiving ileocolonic resection (ICR), there were significant differences in microbiota characteristics between patients with recurrence and patients with first-time surgical resection. The levels of *Bacteroidia*, *Bacteroidales*, *Bacteroidaceae*, and *Bacteroides* were higher in patients with first-time surgical resection (30). On the other hand, Richard T. Liu et al. found that the microbiome of young Americans with major depression was significantly different from that of healthy controls. The levels of *Firmicutes*, *class Clostridia*, and *order Clostridiales* in depressed subjects were lower, while the levels of *Bacteroidia* and *Bacteroidales* in depressed subjects were higher (31). Fabienne Humbel et al. found in their studies on anxiety and depression a negative correlation between psychological distress with the abundance of *Bacteroidia* and *Beta- and gamma-proteobacteria*. In addition, psychological distress was also associated with a decrease in *Bacteroidaceae* and *Desulfovibrionaceae (* 32). Combined with our data, *Bacteroidia* has an important relationship with human diseases.

In addition, the signaling molecules of the gut microbiome are metabolites, which are exchanged between the epithelial surface and the intestinal lumen, enter the circulation, and affect various organs. Veit Rothhammer et al. demonstrated in a mouse model of multiple sclerosis (MS) that metabolites of GM alter the behavior of immune cells such as microglia in the brain and regulate astrocyte activity to promote or prevent inflammation (8). Short-chain fatty acids (SCFA) are the main metabolites of the gut microbiome, which can activate cell receptors and affect cell metabolism. The proven mechanisms include binding with G protein-coupled receptors (GPCRs) and inhibition of histone deacetylase (HDAC) (33). Parts of SCFAs in the circulation can enter the CNS, especially when the blood–brain barrier is breached (26, 34). SCFAs were found to affect the NF-κB pathway in tumor cells and immune cells. The NF-κB pathway affects the production of inflammatory factors such as IL-6 and IL-8 to regulate the tumor microenvironment and promote the development of glioma. SCFAs can also induce Treg differentiation and IL-10 secretion (26). Therefore, further experiments should explore the relationship between *Bacteroidia* and its metabolites with glioma growth through metabolomic analysis.

We have made some strong findings. However, our study still has some limitations. First of all, we did not find a relationship between GM and glioma survival rate, which may be due to the characteristics of subjects and the limited sample size of this study. The clinical factors of human gut microbiome and glioma treatment factors will be considered in the future. In the meantime, both cohort and follow-up studies are needed to investigate possible clinical associations. Secondly, our experiments did not cover all the immune factors in the glioma microenvironment. In the future, it is necessary to study the relationship between GM with immune cells and immune factors in the glioma microenvironment more extensively and deeply and find out the specific regulatory mechanism. Finally, the 16S rDNA analysis is helpful in providing information on the classification of GM. However, metagenomic and metabolomic analyses may shed more light on changes in gut microbiome classification data and help to screen out bacteria or bacterial metabolites that have some utility in glioma development.

Our study on GM and glioma development does not cover a wider range of immune factors. However, our experimental data proved that the GM of mice can regulate Foxp3 expression in the glioma microenvironment and affect the development of glioma. Besides, studies have shown that the gut microbiome can adjust the natural immunity to affect the growth of glioma. These results together support the existence of a functional gut–immune–brain axis. In addition, we speculated that *Bacteroidetes* may affect the growth of glioma, which can provide a new idea for the adjuvant therapy of glioma. The most common therapies of glioma include surgery, radiotherapy, and chemotherapy. Although the immunotherapy for other cancers has developed rapidly, there are currently still no immunotherapies for glioma approved by FDA. Glioma is one of the most immunosuppressive solid tumors. There are many potential relationships between the immune microenvironment of glioma and gut microbiome that need to be verified. Studying the composition of the gut microbiome can provide insights into the immunotherapy of glioma. New perspectives on the therapy of glioma may depend on understanding how the gut microbiome influences its development. At the same time, deciphering the gut–immune–brain axis could provide new ideas for precision medicine and new directions for basic and clinical sciences of human health.

## Conclusion

This study demonstrates the relationship between gut microbiome and glioma in mice. We demonstrated that glioma development induced a decrease in the abundance of *Bacteroidia* in the mouse gut microbiome while it induced an increase in the abundance of *Firmicutes*. In the meantime, we demonstrated that gut microbiome dysbiosis can downregulate Foxp3 expression in mouse brain and promote glioma growth. Maintaining the balance of the gut microbiome microenvironment can upregulate Foxp3 expression in mouse brain and delay glioma development. In addition, we found that the abundance of *Bacteroidia* in the gut microbiome of mice may affect the growth of glioma. Further studies are needed to explore the regulation mechanism of the gut–immune–brain axis on glioma, so as to address the role of the gut microbiome in the development of glioma.

## Data Availability Statement

The raw data supporting the conclusions of this article will be made available by the authors, without undue reservation.

## Ethics Statement

The animal study was reviewed and approved by the Animal Ethics Committee of Zhujiang Hospital of Southern Medical University.

## Author Contributions

YF: conceptualization, methodology, formal analysis, investigation, writing—original draft. QS: software, resources. JC: data curation. YW: project administration. SH: project administration, funding acquisition. All authors contributed to the article and approved the submitted version.

## Conflict of Interest

The authors declare that the research was conducted in the absence of any commercial or financial relationships that could be construed as a potential conflict of interest.

## Publisher’s Note

All claims expressed in this article are solely those of the authors and do not necessarily represent those of their affiliated organizations, or those of the publisher, the editors and the reviewers. Any product that may be evaluated in this article, or claim that may be made by its manufacturer, is not guaranteed or endorsed by the publisher.
